# Vicarious Resilience in Hospital Nurses: A Concept Analysis

**DOI:** 10.3390/healthcare13182279

**Published:** 2025-09-12

**Authors:** Miri Jeong

**Affiliations:** Department of Nursing, Joongbu University, Geumsan-gun 32713, Republic of Korea; miri000517@gmail.com

**Keywords:** concept analysis, vicarious resilience, resilience, hospital nurse, nursing

## Abstract

**Background/Objectives**: Vicarious resilience is a positive psychological transformation in hospital nurses who witness the recovery and strength of patients. This study clarifies the concept using Walker and Avant’s method, identifying defining attributes, antecedents, and consequences to inform theory and practice. **Methods**: A systematic review of Korean and international databases included 21 studies. Through eight analytical steps, four defining attributes were identified: emotional growth, meaning-making, patient-centered engagement, and professional identity reinforcement. **Results**: Key antecedents include empathic exposure and reflective capacity. Consequences are enhanced resilience, reduced burnout, increased job satisfaction, and sustainable caregiving. Unlike compassion satisfaction, post-traumatic growth, or vicarious post-traumatic growth, vicarious resilience stems from relational experiences of patient recovery. The Vicarious Resilience Scale was reviewed, but a hospital nurse-specific measure is needed. **Conclusions**: This analysis offers a foundation for nursing education, organizational support, and interventions to foster emotional sustainability and professional growth in high-stress clinical settings.

## 1. Introduction

Vicarious resilience, first introduced in January 2007 by Hernandez et al. [1], is defined as a positive psychological transformation that occurs in professionals such as therapists, nurses, and first responders as they witness and engage with the resilience demonstrated by trauma survivors [1,2,3]. It was initially conceptualized within the domain of psychotherapy, grounded in empirical observations that trauma therapists frequently experience personal growth, inspiration, and emotional strengthening through their clinical engagement with survivors [1,4]. Over time, the scope of this construct has been expanded and its application extended to a more extensive range of helping professions, beyond the confines of psychotherapy [5,6,7]. Indeed, it has emerged as a vital framework for understanding how healthcare professionals, particularly nurses, derive strength and achieve growth by witnessing their patients’ recovery from trauma and illness [2,8]. Vicarious resilience differs from traditional resilience by its relational origin—stemming not from personal adversity but from engaging with the resilience of others [1]. In hospital settings, where nurses routinely encounter patient suffering and recovery, the potential for vicarious resilience is particularly salient [2,6]. Despite the prevalence of stress and burnout in clinical nursing, many nurses report transformative experiences through close involvement in patient care that reinforce their emotional well-being and professional identity [8].

Resilience refers to the capacity to flexibly respond and adapt when faced with adversity. Although this ability is inherent in all individuals and can be enhanced by intentional effort and training, its degree varies from person to person [2,9,10]. Cooper et al. conducted a concept analysis of nurse resilience, identifying its core attributes as social support, self-efficacy, work–life balance and self-care, humor, optimism, and realism [10]. Previous studies have revealed that resilience enhances nurses’ coping abilities and is associated with reductions in anxiety, depression, and burnout [11,12].

Although vicarious resilience shares conceptual space with constructs such as compassion satisfaction (CS), vicarious post-traumatic growth (VPTG), and post-traumatic growth (PTG), it remains a theoretically distinct phenomenon. CS refers to the emotional fulfillment derived from providing care to others, and it is commonly associated with professional pride and psychological well-being [13,14]. In contrast, PTG denotes positive psychological transformation following direct exposure to traumatic experiences [15], while VPTG describes a similar process of growth that occurs indirectly through empathic engagement with another’s trauma [16,17]. Recent empirical findings in nursing contexts suggest that even in the absence of direct trauma exposure, healthcare professionals may exhibit emotional resilience and derive professional gratification through vicarious experiences of patient recovery [1,18]. Unlike VPTG, which is primarily characterized by cognitive restructuring arising from secondary exposure to trauma narratives, vicarious resilience stems from empathically witnessing recovery and strength. This process fosters relational depth, emotional maturation, and the reinforcement of professional identity [1]. As such, it should be understood as a discrete construct grounded in relational recovery rather than in trauma exposure or general caregiving satisfaction [8].

Nurses in hospital environments are frequently exposed to intense emotional situations, including life-threatening illness, patient deterioration, and end-of-life care [2,19]. Although such experiences can be emotionally draining, they may also generate psychological growth, particularly when nurses witness patients overcoming major health challenges [2,20]. Unlike mental health nurses, whose roles emphasize long-term therapeutic relationships, hospital nurses typically work in fast-paced, high-pressure environments [6]. In the Korean context, the term “hospital nurses” in this study denotes staff working in non-psychiatric acute and specialized wards of general hospitals (e.g., medical–surgical, ICU, oncology), whereas mental health nurses may be hospital-based in psychiatric wards or community-based in mental-health services. This clarification underscores the relevance of vicarious resilience, and it helps distinguish it from adjacent constructs in psychiatric contexts [2,20,21]. Accordingly, we examine how vicarious resilience manifests in hospital nursing and how it differs from related constructs such as CS and burnout-related processes [7,22].

A concept analysis is essential to clearly define vicarious resilience in hospital nurses, as the term is often used inconsistently across disciplines [6,7]. This study aims to clarify the concept of vicarious resilience in hospital nurses by identifying its defining attributes, antecedents, and consequences using Walker and Avant’s concept analysis method, thereby informing theoretical development and practical nursing interventions [20,22]. Accordingly, a new conceptual perspective is introduced to capture the positive psychological transformation that hospital nurses experience by witnessing patients’ recovery and resilience.

Concept analysis is defined as a systematic process aimed at exploring the structure and function of a given concept by closely examining its fundamental components [6,10,22]. This approach incorporates evidence derived from diverse academic disciplines to clarify the meaning of the concept and develop an operational definition through the structured representation of conceptual knowledge and data analysis methods [23]. The implementation of a methodological framework of this nature serves as an effective strategy to integrate heterogeneous bodies of knowledge, thereby contributing to the expansion of theoretical foundations and the enhancement of conceptual clarity [23].

In summary, this study aims to conduct a conceptual analysis of vicarious resilience among hospital nurses and delineate its defining attributes and theoretical definition. The conceptual analysis method proposed by Walker and Avant is employed to clarify the meaning of the concept by identifying its essential characteristics, antecedents, and consequences. The objective of this analysis is twofold: first, to promote consistent understanding and communication among researchers investigating this phenomenon and, second, to establish a theoretical foundation for the development of measurement instruments and evidence-based nursing interventions.

## 2. Methods

Walker and Avant’s concept analysis method has been widely utilized in nursing research to clarify poorly defined or conceptually ambiguous terms. This study applies the same framework to analyze the concept of vicarious resilience as experienced by hospital nurses [23]. Walker and Avant proposed an eight-step process for concept analysis: (1) selecting the concept of interest, (2) determining the aims or purposes of analysis, (3) identifying all discoverable uses of the concept, (4) determining the defining attributes, (5) constructing a model case, (6) constructing additional borderline, related, and contrary cases, (7) identifying antecedents and consequences, and (8) defining empirical referents [24].

### 2.1. Selecting the Concept and the Purpose of the Analysis

The literature review included both dictionary definitions and conceptual uses of vicarious resilience across academic disciplines. The search period spanned studies published from January 2007 to May 2025. The inclusion criteria were as follows: (1) peer-reviewed academic publications and (2) articles written in either Korean or English. The exclusion criteria were as follows: (1) grey literature, including dissertations, conference abstracts, magazines, and books; (2) articles that did not provide identifiable attributes of the concept; and (3) publications without accessible full text.

**Figure 1:**
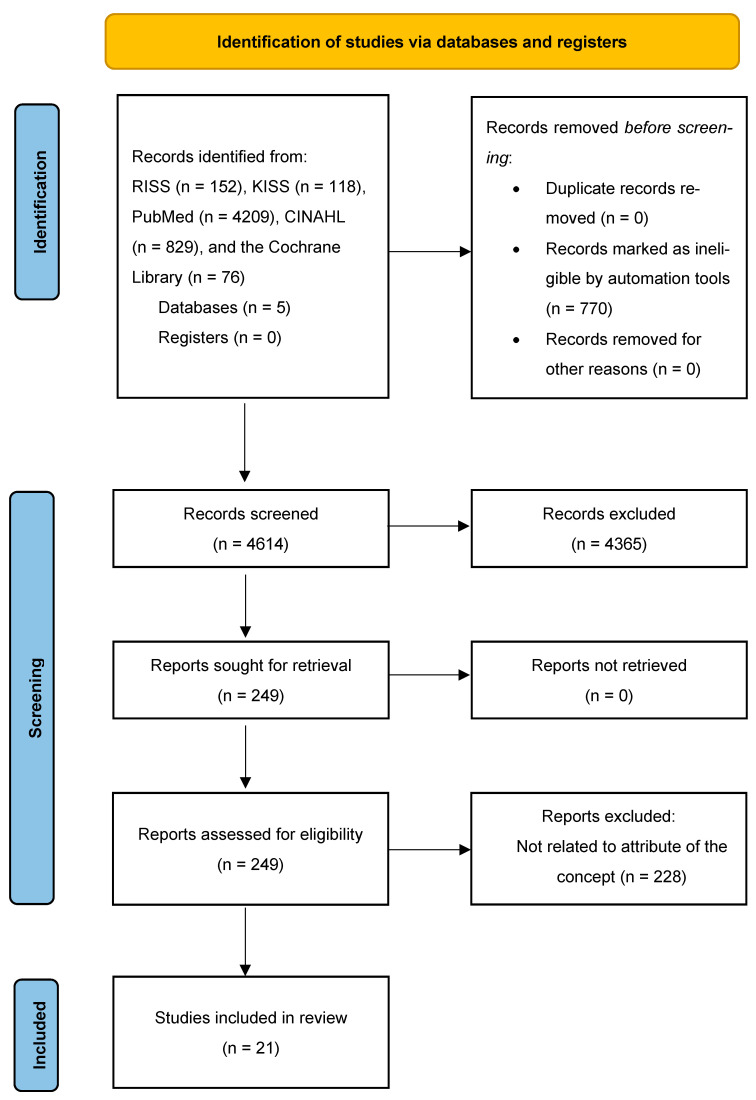
Flow chart of study selection.

We followed PRISMA 2020 [24] and searched five bibliographic databases—RISS, KISS, PubMed, CINAHL, and the Cochrane Library. Search strings combined controlled vocabulary and keywords related to vicarious or indirect forms of resilience and nursing, using the Boolean operators AND/OR (e.g., “vicarious resilience,” “secondary resilience,” “resilience,” “compassion satisfaction,” “post-traumatic growth,” “vicarious post-traumatic growth,” “nurs*,” “nursing care”). Across the databases, we identified 5384 records in total (RISS: 152; KISS: 118; PubMed: 4209; CINAHL: 829; Cochrane Library: 76). No trial registers or other sources were searched. Before screening, 770 duplicate or ineligible records were removed. We screened 4614 titles/abstracts and excluded 4365. We sought and retrieved the full texts of 249 reports (0 not retrieved) and assessed 249 reports for eligibility. Of these, 228 were excluded after reviewing the full text, leaving 21 studies included in the review (7 Korean-language and 14 international) (Figure 1).

### 2.2. Researcher Preparation

The author brings extensive clinical experience in acute and post-acute care settings, providing deep insight into the study population’s needs. As part of a doctoral program, the author completed coursework on nursing concept development, including a comprehensive literature review utilizing Walker and Avant’s concept analysis method. Additionally, the author has engaged in diverse nursing research projects, collaborating with interdisciplinary teams to explore clinical practices and outcomes. Participation in academic conferences and professional development workshops has further strengthened the author’s expertise in conceptual inquiry and methodological rigor, ensuring a robust foundation for this study.

## 3. Results

### 3.1. Dictionary Definitions in Hospital Nursing

According to the Cambridge Dictionary, the term vicarious refers to experiences gained by observing or reading about others rather than by directly engaging in the activities. Similarly, Merriam-Webster defines vicarious as something “experienced or realized through imaginative or sympathetic participation in the experience of another.” The Cambridge Dictionary [25] defines resilience as “the ability to be happy, successful, etc. again after something difficult or bad has happened,” while Merriam-Webster [26] describes resilience as “the ability to recover from or adjust easily to adversity or change,” also noting a secondary meaning related to physical elasticity.

Hospital nurses are defined as trained healthcare providers who deliver direct care to individuals suffering from illness or injury within hospital settings. The Cambridge Dictionary [25] defines a nurse as someone whose job is to care for people who are ill or injured, especially in a hospital, while Merriam-Webster [26] defines a nurse as a professionally trained person who provides care for the sick or infirm, particularly in clinical environments. Expanding upon these definitions, the International Council of Nurses defines nurses as essential contributors within complex clinical systems, providing person-centered and evidence-based care that supports not only acute medical treatment but also long-term recovery and the overall well-being of patients [27]. Based on these combined dictionary definitions, vicarious resilience in hospital nurses can be defined as the ability to recover from or adapt to adversity or change, acquired by nurses who care for the ill or injured in hospital settings through indirect or empathic experiences of their patients’ resilience.

### 3.2. Conceptual Use in Other Disciplines

#### 3.2.1. Psychology

In the field of psychology, vicarious resilience is defined as the positive psychological transformation that occurs in professionals such as therapists by empathically engaging with trauma survivors who demonstrate resilience [1,4]. Unlike vicarious trauma, which causes negative emotional consequences, vicarious resilience fosters positive cognitive shifts, a renewed sense of purpose, enhanced emotional strength, and professional growth. Key elements include witnessing client resilience, emotional attunement, reflective practice, meaning-making, and professional connectedness. Psychological research has highlighted that this construct not only mitigates the effects of burnout and secondary trauma but also promotes well-being and sustainability in trauma-related professions [1,4].

#### 3.2.2. Educational Psychology

In educational psychology, vicarious resilience refers to the positive emotional and professional growth experienced by teachers or educators through observing the resilient behaviors and narratives of their students. This phenomenon is particularly pronounced in challenging educational environments, such as under-resourced schools or communities affected by adversity, where educators witness students overcoming significant obstacles [28]. The process fosters hope, enhances self-efficacy, and strengthens relational skills, enabling educators to reconstruct their personal and professional contexts. This construct also supports educators in fostering a positive classroom culture that values resilience and mutual support.

#### 3.2.3. Social Work

In the field of social work, vicarious resilience is conceptualized as the positive personal transformation experienced by practitioners through witnessing the growth and recovery of their clients. This process is particularly relevant for social workers supporting individuals overcoming trauma, adversity, or systemic challenges [29]. By engaging with clients’ stories of resilience, social workers develop enhanced empathy, new insights into human strength, and increased emotional resilience, which contribute to their professional sustainability [29]. Key mechanisms include reflective supervision and peer support, which allow social workers to process and integrate the positive transformations they observe [30]. Additionally, vicarious resilience fosters a sense of professional connectedness, as social workers share strategies and insights gained from their clients’ resilience, further reinforcing their commitment to advocacy and social justice.

Beyond the helping professions, emerging applications in criminology and art therapy suggest a broader interdisciplinary reach. In these domains, vicarious resilience is an emergent construct observed among practitioners who witness resilience in clients navigating justice systems or expressing recovery through creative processes. Nevertheless, the construct remains underdefined in these contexts, and its field-specific mechanisms and contributing factors are insufficiently articulated [31,32].

### 3.3. Conceptual Use in the Nursing Literature

Alotaibi (2024) systematically analyzed the concept of vicarious resilience within the context of mental health nursing, defining it as the positive psychological transformation that mental health nurses experience by witnessing the resilience and growth of their patients [6]. Through concept analysis, the study identified the term’s defining attributes: empathy, hope, resourcefulness, awareness, and spirituality. These attributes are supported by antecedents such as engaging with patient trauma narratives, practicing self-awareness and self-care, and receiving peer support. The analysis revealed that vicarious resilience can lead to enhanced emotional well-being, a redefined life purpose, improved adaptability, personal growth, and increased individual resilience [6]. Alotaibi concluded that understanding and applying the concept of vicarious resilience has significant implications for developing nursing interventions, supporting professional well-being, and advancing theory in mental-health nursing practice.

Similarly, perinatal palliative care professionals often experience emotional challenges such as moral distress and burnout owing to high-stakes clinical encounters [2]. However, this study identifies vicarious resilience as a positive outcome—defined as personal growth and psychological renewal—resulting from witnessing patients’ resilience in the face of life-limiting fetal diagnoses. Key elements facilitating this process include authentic debriefing sessions, peer mentoring, manageable caseloads, robust self-care practices, relational efficacy, and cultural and spiritual humility—factors that foster personal resilience, ultimately contributing to healthcare professionals’ well-being and sustained ability to provide compassionate care [2]. The authors conclude that vicarious resilience requires strategic, multifaceted institutional support, not merely individual efforts, to strengthen resilience across perinatal palliative care teams.

#### 3.3.1. Identification of the Concept’s Defining Attributes

We conducted this concept analysis of vicarious resilience in hospital nurses using Walker and Avant’s [23] method, through which we identified four defining attributes:

1. Emotional growth: Emotional growth is the emotional development that nurses experience by witnessing patient recovery journeys. It reflects a deepened emotional capacity and maturity gained from empathic encounters in clinical settings [5,33,34,35,36].
2. Meaning-making: Nurses reinterpret and reframe the meaning of their clinical roles and caregiving work through the experiences of patient recovery, fostering a renewed sense of purpose [34,35,37,38,39].
3. Patient-centered engagement: Active involvement in the patient’s recovery journey includes emotional investment, empathic listening, and therapeutic presence. This fosters a deep connection between the nurse and the patient [34,38,39,40].
4. Professional identity reinforcement: The nurses’ professional identity and mission are strengthened, leading to increased pride, purpose, and motivation in their roles [37,41,42,43].

#### 3.3.2. Constructing Cases

According to Walker and Avant, identifying a model case, as well as additional types of cases, is a useful strategy to clarify the defining attributes of a concept [24]. This paper presents model, related, borderline, and contrary cases of vicarious resilience as experienced by hospital nurses, as summarized in Table 1.

### 3.4. Identification of Antecedents and Consequences of the Concept

#### 3.4.1. Antecedents

The development of vicarious resilience among hospital nurses is facilitated by several key antecedents, primarily grounded in their empathic engagement with patients. Empathic exposure is the foundational condition under which vicarious resilience is likely to emerge [37,40]. It involves nurses’ deep emotional attunement to patient experiences, often encompassing both suffering and recovery. Three primary subcomponents characterize this exposure. First, witnessing patient recovery or decline entails observing patients as they navigate physical and emotional adversity, whether through recovery or the dignified process of deterioration and death [34,37,38,39,40,41,44,45], and it serves as critical moments of meaning-making and personal insight. Second, life-saving or end-of-life interventions create intense clinical situations that heighten emotional engagement [9,35,38,39,44], allowing for deep relational connection and existential reflection, both of which may foster resilience. Third, frequent emotional engagement refers to the consistent and intimate emotional interactions nurses maintain with patients as part of routine care, which cumulatively contribute to empathic depth and personal transformation [35,38]. The second core antecedent is reflective capacity, which enables nurses to process, interpret, and integrate emotionally charged clinical experiences. This internal process of reflection allows them to derive meaning from adversity, and it includes personal reflection and self-awareness—through which nurses evaluate their emotional responses and coping mechanisms [34,35,36,37,39,46,47]—and team-based or peer support, with collaborative reflection and shared emotional processing in a supportive team context enhancing psychological resilience [34,36,41,42,43,46].

#### 3.4.2. Consequences

The consequences of vicarious resilience in hospital nurses are both intrapersonal and organizational in nature. They extend beyond individual well-being and influence broader dimensions of workforce sustainability and care quality.

Foremost among these is resilience enhancement, whereby nurses develop greater psychological stamina and flexibility because of repeated exposure to patient recovery and subsequent emotional processing. This enhancement equips nurses to cope more effectively with occupational stressors and adapt to the emotional volatility inherent in clinical practice [5,34,35,37,39,46,48]. Another salient outcome is burnout buffering, whereby nurses demonstrate reduced susceptibility to compassion fatigue and emotional exhaustion. The positive emotional reinforcement derived from witnessing patient recovery may serve as a psychological counterbalance to the otherwise deleterious effects of caregiving in high-stakes environments [33,34,35,37,39,45,49]. Job satisfaction is another frequently reported outcome. Nurses who derive meaning and purpose from their work—particularly through transformative experiences with patients—are more likely to feel fulfilled and committed to their roles. This satisfaction reinforces their professional identity and may contribute to greater engagement in patient care [37,38,39,41,43,48,50]. Lastly, sustainable caring is a broader organizational consequence. The internal resources built by vicarious resilience support long-term career sustainability by reducing turnover intentions and promoting retention among experienced nursing staff [42,51]. As shown in Table 2, constructed cases were developed to clarify the defining attributes of vicarious resilience in hospital nurses.

### 3.5. Empirical Referents

Empirical referents are observable indicators that are tied to the defining attributes of a concept rather than being tied to the concept as a whole [23]. A representative instrument is the Vicarious Resilience Scale (VRS), originally developed by Killian et al. to capture positive changes among trauma professionals working with survivors [52]. Overlaps are evident with emotional growth, meaning-making, and patient-centered engagement; however, professional identity reinforcement from hospital nursing practice is not explicitly represented in the VRS. As of August 2025, the VRS is established in English per the original development paper, and formal cross-language psychometric validation specific to the VRS appears limited. Accordingly, translating, culturally adapting, and validating the VRS for Korean hospital nurses—or developing a context-specific measure—is warranted.

The VRS comprises seven subdomains that assess positive psychological changes experienced by trauma professionals by witnessing client resilience [52]. Among the defining attributes identified in this study—namely, emotional growth, meaning-making, and patient-centered engagement—conceptual overlaps with the VRS are evident. However, the attribute of professional identity reinforcement, derived from the specific context of hospital nursing practice, is not explicitly reflected in the VRS. This warrants the development of a new measurement instrument tailored to the experiential and contextual features of hospital nurses.

## 4. Discussion

This study analyzed the concept of vicarious resilience in hospital nurses, identifying its defining attributes, antecedents, and consequences. Vicarious resilience is conceptually distinct from general resilience or PTG, and it refers to the positive emotional and professional transformations experienced by nurses after witnessing patient recovery processes [1]. This concept is particularly applicable in clinical settings such as intensive care, palliative care, and oncology wards, where patient suffering and recovery frequently coexist [2,11,12].

The four defining attributes—emotional growth, meaning-making, patient-centered engagement, and reinforcement of professional identity—represent a deep, transformative process that strengthens nurses’ internal and professional selves [5,6]. Observing patient resilience, empathizing with their experiences, and engaging in reflective introspection contribute to professional identity reinforcement and extend beyond emotional labor [4,13]. This suggests that vicarious resilience is a sustainable emotional and occupational resource rather than a temporary reaction [10,37].

Although vicarious resilience shares similarities with CS, PTG, and VPTG, it is distinct [15,16]. CS focuses on positive emotions from caregiving, while vicarious resilience emphasizes empathic engagement with patient recovery [14,39]. PTG centers on personal trauma recovery, whereas vicarious resilience highlights growth from witnessing the recovery of others [17,18]. This study provides a theoretical basis for this underexplored internal transformation in clinical nursing practice [7,20].

This study builds on Alotaibi’s concept analysis of vicarious resilience in mental health nursing (24 articles, attributes: empathy, hope, resourcefulness, awareness, spirituality) [6]. Our analysis (21 studies) focuses on hospital nurses in acute care settings, identifying emotional growth, meaning-making, patient-centered engagement, and professional identity reinforcement. These reflect the fast-paced acute care context, contrasting with the long-term therapeutic relationships in mental health nursing. While Alotaibi emphasized spirituality, our findings prioritize relational and professional dimensions, suggesting that spirituality may be less prominent in general hospital settings, although it merits further exploration.

Vicarious resilience also contributes to understanding the vicarious traumatization experiences of nurses. By witnessing patient recovery, nurses transform potentially negative empathic exposure into positive growth, mitigating emotional exhaustion and fostering resilience that buffers against burnout in high-stress settings [3,33]. This counterbalance supports emotional sustainability and coping.

Incorporating the Korean and international literature, this study finds similarities in vicarious resilience experiences such as empathic engagement and reflection across global contexts. However, Korea’s spiritual traditions (e.g., Buddhism, Confucianism, Christianity) may influence meaning-making. While spirituality was not a core attribute—likely due to the acute care focus and limited emphasis in the reviewed literature—Korean studies suggest its relevance in palliative care [19,40]. For instance, spiritual humility in palliative settings [2] may intersect with meaning-making. Its absence as a primary attribute reflects the empirical focus on acute care, but future research should explore the role of spirituality in vicarious resilience, particularly in culturally diverse regions such as Korea, to identify potential differences in coping mechanisms.

The concept varies across disciplines. Psychology emphasizes the transformation of therapists [4,28], while educational psychology and social work focus on structural or collective resilience [10,29]. Within nursing, the experiences of psychiatric nurses differ from those in acute care [6,33]. This study establishes vicarious resilience as an independent, practice-oriented concept for hospital nurses, underscoring the need for theoretical delineation [7,21].

From a clinical perspective, vicarious resilience has significant implications for nursing education and leadership. Hospital nurses face emotionally taxing situations like end-of-life care and chronic illness management [44,45]. The antecedents—empathic exposure and reflective capacity—show that these environments can foster positive psychological resources [40,41]. However, leveraging vicarious resilience requires targeted strategies. In nursing education, integrating vicarious resilience into curricula can reframe emotional labor as a growth opportunity. For example, case-based learning, reflective journaling, or simulation exercises can help students and novice nurses develop reflective capacity, fostering professional identity formation. Educators must critically balance the teaching of empathic engagement with preventing emotional overload, as unsupported over-engagement risks burnout [3,33]. Curricula should include structured reflection modules to equip students for high-stress settings without idealizing emotional labor.

For nursing leadership, vicarious resilience informs strategies to enhance workforce sustainability. Leaders should implement systemic interventions like regular team debriefings, peer mentoring programs, and empathy-based training to support reflective capacity and emotional engagement [34,43]. A critical challenge is addressing organizational constraints, such as understaffing or time pressures, which can undermine resilience-building efforts [9]. Leaders must advocate for policy changes such as workload adjustments or dedicated reflection time to foster cultures prioritizing resilience. Leadership training should focus on modeling empathic behaviors and creating psychologically safe environments for emotional processing, enhancing care quality and nurse retention. Without systemic support, the potential for vicarious resilience may remain underutilized.

Future research should quantify vicarious resilience and explore its applicability across nursing contexts. Investigating the role of spirituality, especially in culturally rich settings such as Korea, could reveal nuanced coping mechanisms. Organizational interventions, such as team debriefings and empathy-based programs, should be evaluated for effectiveness in fostering vicarious resilience.

The conceptualization of vicarious resilience offers a practical resource to enhance nurses’ emotional recovery, job satisfaction, and caregiving sustainability. It shifts the focus from compassion fatigue and burnout to positive transformation within healing relationships. First, it provides a theoretical foundation for strategies to strengthen psychosocial resources through education and leadership initiatives. Second, in nursing education, it should be integrated into professional identity formation, framing emotional labor as an opportunity for growth. Third, in nursing management, it guides policy development to create supportive organizational cultures, ensuring systemic changes that amplify resilience. By focusing on intrapsychic changes from relational dynamics, this study aligns with the interaction-centered paradigm of nursing science and reinforces its conceptual identity.

## 5. Conclusions

This concept analysis defines vicarious resilience in hospital nurses as a dynamic process of positive psychological transformation marked by emotional growth, meaning-making—including, where appropriate, spiritual reflection—patient-centered engagement, and reinforcement of professional identity. It arises indirectly through empathic exposure to patient recovery, and it is consolidated through deliberate reflection and consistent peer support. Vicarious resilience is distinct from compassion satisfaction, post-traumatic growth, and vicarious post-traumatic growth because it develops through relational experience and directly strengthens the nurse’s professional identity. As a sustainable psychological resource, it mitigates burnout, enhances job satisfaction, and supports sustainable caregiving. These findings justify the integration of vicarious resilience into nursing education through structured reflective practices and routine debriefings that promote emotional processing and professional growth, particularly for novice nurses, and that reframe emotional labor as an opportunity for development. Healthcare institutions should also establish structured peer-support systems and culturally informed resilience training that reflect spirituality and collectivist values relevant to Korean nursing practice. We further recommend the development and validation of nurse-specific measurement tools that capture professional identity and culturally grounded meaning-making. Future research should quantify the emergence and impact of vicarious resilience across diverse clinical settings and cultural contexts to inform evidence-based interventions and to build sustainable workforce models that promote nurse well-being and patient-care quality.

## Figures and Tables

**Table 1:** Literature mapping of vicarious resilience components in hospital nurses.

**Antecedents**	**Reference Number**
Empathic exposure	
Witnessing patient recovery or decline	[34,37,38,39,40,41,43,44,45]
Life-saving or end-of-life interventions	[9,35,39,40,44]
Frequent emotional engagement	[35,38]
Reflective capacity	
Personal reflection and self-awareness	[34,35,37,38,39,46,47]
Team-based or peer support	[34,35,41,42,43,46]
**Attributes**	**Reference Number**
Emotional Growth	[5,33,34,35,36]
Meaning-Making	[34,35,37,38,39]
Patient-Centered Engagement	[34,36,38,40]
Professional Identity Reinforcement	[37,41,42,43]
**Outcomes**	**Reference Number**
Resilience Enhancement	[5,34,35,37,39,46,48]
Burnout Buffering	[33,34,35,37,39,45,49]
Job Satisfaction	[37,38,39,41,43,48,50]
Sustainable Caring	[42,51]

**Table 2:** Constructed cases to analyze the concept of vicarious resilience in hospital nurses.

Cases and Their Definitions	Example	Analysis
Model case		
A model case represents a complete and exemplary instance of a concept, illustrating all of its defining attributes. It may be drawn from actual clinical practice, derived from the existing literature, or constructed by the researcher. The following model case was developed by the author based on the defining attributes identified in this study.	Nurse Kim is an experienced clinical nurse who has worked in the intensive care unit for ten years. One day, he was assigned to care for a male patient in his twenties who had been admitted due to multiple traumatic injuries. The patient underwent repeated surgeries and long-term ventilator support, hovering between life and death. However, with strong willpower and the support of his family, he gradually began to recover. Nurse Kim closely observed and empathized with the patient’s pain, fear, and hope while providing essential and life-sustaining care every day. Through this experience, he developed a profound sense of awe regarding human resilience, and the patient’s transformation resonated with him as if it were his own emotional shift. Later, he shared the recovery story with fellow nurses and took time for self-reflection, processing the meaning of his emotional experiences. He remarked, “Through this patient, I was reminded not only of the value of life, but also of why I chose this profession.” As a result, Nurse Kim began to engage in more vibrant empathy and provide care with a renewed focus on each patient’s individuality. His sense of professional identity was also significantly strengthened.	This case exemplifies a prototypical instance of vicarious resilience in hospital nurses, as it fully embodies the defining attributes of the concept—empathic exposure, reflective capacity, emotional growth, meaning-making, patient-centered engagement, and professional identity reinforcement. Furthermore, it consistently manifests the anticipated consequences, including enhanced resilience, buffering against burnout, increased job satisfaction, and the promotion of sustainable caregiving practices.
Borderline case		
A borderline case refers to an example that includes most, but not all, of the defining attributes of the concept. The following borderline case was constructed by the author and includes only two attributes: empathic exposure and emotional connection accompanied by the gratification of caregiving.	Nurse Choi is a second-year novice nurse working in a rehabilitation ward. One day, he heard from a colleague about a patient with severe brain injury who had regained consciousness and made a significant recovery through rehabilitation. The story brought great joy and hope to the entire team, and Nurse Choi also felt a sense of inspiration, thinking, “Our team is truly doing meaningful work.” The case momentarily led him to reflect on the value of his profession, and he even considered sharing the story with his nursing school juniors. However, Nurse Choi had no direct interaction with the patient involved, and his emotional response remained indirect and temporary. Although he was touched by the story, it did not lead to any concrete changes in his nursing practice or emotional attitude. The experience did not contribute to deeper self-reflection, job satisfaction, or professional growth. Soon afterward, he became immersed in his daily responsibilities, and the emotional impression faded into the routine of his work life.	This case is considered a borderline example of vicarious resilience, as it lacks fulfillment of the core attributes, such as sustained empathic exposure, reflective capacity, and enduring emotional transformation. Although emotional responses were observed, the essential conceptual components of vicarious resilience were not clearly manifested.
Related case		
A related case is one that closely resembles the concept under analysis, but ultimately reflects a different underlying idea. Through careful examination, it becomes possible to distinguish which defining attributes of the target concept are present and which are not. The following related case was constructed by the author.	Nurse Lee volunteered to work in a designated COVID-19 ward during the initial outbreak of the pandemic. For several days and nights, he cared for critically ill patients, enduring the relentless physical strain of working in protective gear and the emotional toll of witnessing continuous deaths. Over time, he began to feel emotionally numb. One day, an elderly patient who had been in critical condition began to recover and, for the first time, spoke, saying, “I want to go home alive.” The nurse was deeply moved by this moment and began to follow the patient’s recovery process closely, holding it in his heart. Despite this emotional resonance, his relationship with the patient remained limited, and it lacked the depth of the emotional engagement typically associated with vicarious resilience. Instead of forming a strong empathic connection, the nurse was more preoccupied with reflecting on his own intense stress and fear. After his assignment ended, he enrolled in a counseling program, during which he stated, “That experience made me realize how vulnerable I was—and how much I need to be thankful.” Following this, he focused on rebuilding relationships with his family and slowing down to take care of himself. Although the nurse witnessed the patient’s recovery, the experience did not become internalized through emotional interaction or a caregiving relationship. The primary catalyst for his change was the trauma he personally endured, and his response centered on overcoming that trauma. The transformation he experienced led to a broader shift in life perspective rather than a specific change in clinical nursing practice or professional identity.	The case of the nurse presented in this study illustrates the concept of post-traumatic growth. Both vicarious resilience and post-traumatic growth involve psychological development and the reconstruction of meaning. However, vicarious resilience emerges indirectly through witnessing the recovery of others, whereas post-traumatic growth results from personal experiences of trauma and the subsequent individual recovery process.
Contrary case		
A contrary case is a clear example of what the concept is not, in which none of the identified defining attributes are present. The following contrary case was constructed by the author.	Nurse Jeong has been working in the emergency department for five years and has recently reported symptoms of emotional exhaustion and burnout. Faced daily with cases involving suicide attempts, severe trauma, and abuse, he had become desensitized, performing his tasks in a mechanical manner without a sense of purpose or an awareness of their impact on patient outcomes. When a teenage patient was brought in following a self-harm incident, Jung emotionlessly checked vital signs and administered medication before quickly moving on to the next case. Even when the patient tearfully expressed, "I want to live," Jung responded not with empathy but with a cynical thought, thinking it was just another typical case. He minimized patient interaction and refused to discuss the case with colleagues. When a coworker attempted to share their feelings about the situation, Jung dismissed the conversation by saying that talking about such things only made him more exhausted. His emotional detachment escalated into avoidant behavior, where he even rejected routine expressions of empathy. He frequently expressed dissatisfaction with his job and thoughts of leaving the profession altogether.	This case represents a contrary example to vicarious resilience, exhibiting characteristics that are antithetical to the core attributes of the concept. It is marked by avoidance of empathic exposure, emotional detachment, absence of reflection, lack of meaning-making, weakened professional identity, and diminished resilience.

## Data Availability

The original contributions presented in this study are included in the article. Further inquiries can be directed to the author.

## Abbreviations

The following abbreviations are used in this manuscript:

CS	compassion satisfaction
KISS	Korean Studies Information Service System
RISS	Research Information Sharing Service
PTG	post-traumatic growth
VPTG	vicarious post-traumatic growth
VRS	Vicarious Resilience Scale
